# The Spread of *Helicoverpa armigera* (Lepidoptera: Noctuidae) and Coexistence with *Helicoverpa zea* in Southeastern Brazil

**DOI:** 10.3390/insects8030087

**Published:** 2017-09-04

**Authors:** Fábio A. Pinto, Marcos V. V. Mattos, Farley W. S. Silva, Silma L. Rocha, Simon L. Elliot

**Affiliations:** Department of Entomology, Universidade Federal de Viçosa (UFV), Campus Universitário s/n,Viçosa, MG 36570-900, Brazil; marcos2vmattos@hotmail.com9 (M.V.V.M.); farleyw@gmail.com (F.W.S.S.); silmalrocha22@gmail.com (S.L.R.); selliot@ufv.br (S.L.E.)

**Keywords:** Noctuidae, pest, PCR-RFLP, mitochondrial, DNA

## Abstract

*Helicoverpa armigera*, one of the world’s most destructive crop pests, was first documented in Brazil in 2013. Within a few months, this polyphagous insect had spread over the Northeast and Central-West of Brazil, causing great agricultural losses. With several reports of populations resistant to pesticides and Bt crops around the world, there is great concern about the spread of this pest in Brazil. There is confusion about the actual distribution of this species due to the high morphological similarity with the native corn earworm *Helicoverpa zea*, which may also coexist with *H. armigera* in the field. Our aims here were (i) to confirm its presence in the State of Minas Gerais, one of the most important agricultural regions in the country; and (ii) to assess the co-occurrence of this pest with the congeneric corn earworm *H. zea*. Using molecular screening, we confirmed the presence of *H. armigera* in Bt-crops of soybean and cotton, and non-Bt-crops of soybean, cotton and maize. Mixed infestations of *H. armigera* with *H. zea* were found in non-Bt maize (Viçosa, Southeastern Minas Gerais). These results highlight the need for adequate control strategies for *H. armigera* in Brazil, to deal with its polyphagous feeding habits, high dispersal capacity and possible risks of hybridization with congeneric species.

## 1. Introduction

The old world bollworm *Helicoverpa armigera* (Hübner) (Lepidoptera: Noctuidae) is considered one of the most important agricultural pests in the world. Until recently, this pest was considered absent in the Americas. In 2013, though, it was officially recorded in Brazil [1,2]. Within a few months, the species spread across the Northeast and Central-West of Brazil, causing billions of dollars of losses to the 2012/2013 soybean and cotton harvests [3]. More recently, unidentified noctuids have been reported causing great economic losses in Minas Gerais (one of the principal agricultural regions in Brazil), which raised the possibility that these were *H. armigera* [4].

The presence of *Helicoverpa* species in this region, along with the proximity to other regions with already confirmed infestations, suggests that *H. armigera* may also be present in Minas Gerais. However, specific identification is complex and there is the potential for misidentification due to morphological similarities between *H. armigera* and the corn earworm *Helicoverpa zea* [5]. For these reasons, molecular analysis (for example PCR-RFLP) is necessary in most cases to distinguish these two species [5]. Thus, our aim here was (i) to confirm the presence of *H. armigera* in economically important crops in the Southeast of Brazil and (ii) to assess whether *H. armigera* and *H. zea* co-occur in this region.

## 2. Material and Methods

*Helicoverpa* samples were collected in municipalities from three locations in Minas Gerais (Microregion of Patos de Minas, Alto Paranaíba and Zona da Mata) between February and March 2015. All sampling was authorized by the farmers and the agencies responsible for such collections of biological materials (IBAMA licence no. 15BR017065/DF). Approximately 15 to 30 caterpillars were collected in each sampling event, and these were stored in individual micro-tubes with absolute ethanol. A total of 212 caterpillars were thus collected in 10 different sites, in soybean, cotton and maize crops. Previously identified *H. armigera* laboratory samples were used as positive control for genetic identifications [6].

DNA extractions were performed using Wizard^®^ Genomic DNA Purification (Promega, Madison, WI, USA) following the manufacturer’s protocol for extraction of genomic DNA from plant tissue. An additional RNAse step (Promega, Madison, WI, USA), again following the manufacturer’s protocol, was included in order to remove RNA during isolation of genomic DNA. *Helicoverpa* samples were identified (as *H. armigera* or *H. zea*) with polymerase chain reaction (PCR) by amplifying a 511 base pairs (bp) fragment of Cytochrome c oxidase subunit I (COI) mitochondrial gene using COI-F02 and COI-R02 primers [5]. The PCR conditions were as follows: (1) 94 °C for 5 min, 1 cycle; (2) 94 °C for 30 s, 50 °C for 60 s, 72 °C for 60 s, 35 cycles; and (3) 72 °C for 10 min, 1 cycle.

**Figure 1:**
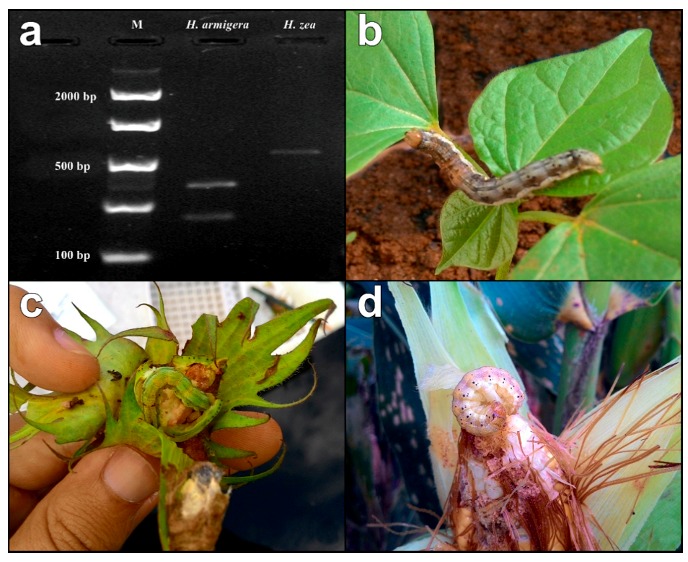
(**a**) Partial Cytochrome c oxidase subunit I (COI, 511 bp) RFLP pattern (digested with BstZ17I) of *Helicoverpa armigera* and *Helicoverpa zea*, with bands separated on 2% agarose Gel. M = DNA ladder; (**b**–**d**) *Helicoverpa* larvae were found attacking leaves and pods in (**b**) soybean; (**c**) leaves, flowers and bulbs in cotton; and (**d**) maize ears. In soybean and cotton crops, only *H. armigera* was found, but in maize both *Helicoverpa* species were found, indicating possibilities of hybridization. For cotton and soybean, *H. armigera* was also found in Bt crops.

In order to distinguish between *H. armigera* and *H. zea*, a 5 µL-aliquot of each amplified PCR product was digested with ten units of restriction enzyme (BstZ17I) in a 20 µL reaction volume according to the manufacturer’s instructions (New England Biolabs, Ipswich, MA, USA). Upon incubation for 6 h at 37 °C, the digested products were separated by electrophoresis and photo-documented under UV transillumination. Within partial COI sequences, a single base pair mutation is present at the BstZ17I recognition site (GAATAC) in *H. armigera*, but not in *H. zea* (GTATAC). Therefore, the restriction endonuclease reaction digests the 511 bp COI PCR product for *H. armigera*, giving two bands of 318 bp and 193 bp, while the fragment for *H. zea* is fully conserved (see Figure 1a; [5]).

## 3. Results and Discussion

**Figure 2:**
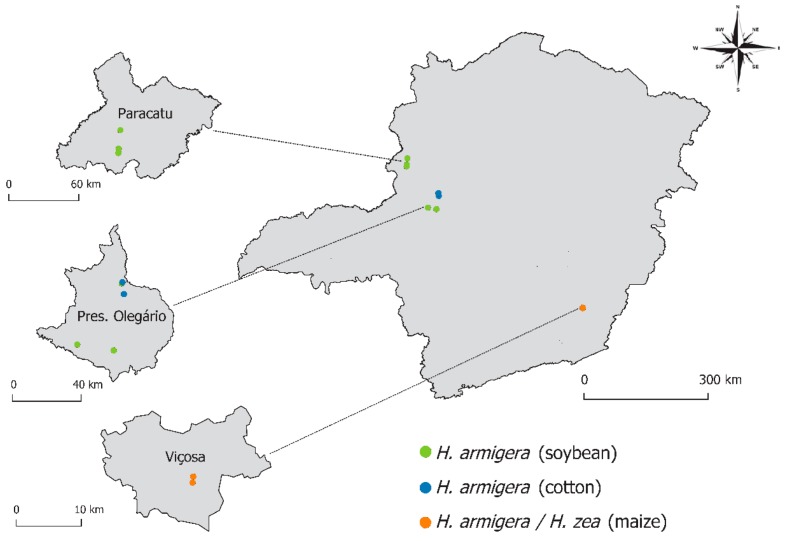
Sampling sites in the State of Minas Gerais where *Helicoverpa armigera* and *Helicoverpa zea* were collected in the study. The ten samplings sites were focused in three municipalities (Highlighted maps: Paracatu, Presidente Olegário and Viçosa). Individuals of *H. armigera* were found in six soybean (green circles) and two cotton (blue circles) sampling sites, including Bt and Non-Bt crops. Mixed infestations between *H. armigera* and *H. zea* were found in two sampling sites with non-Bt maize crop (orange circles).

The restriction enzyme analysis confirmed the presence of *H*. *armigera* at all 10 sampling sites (Figure 2). Individuals identified as *H. armigera* were found in soybean, cotton and maize crops attacking leaves and pods in soybean; leaves, flowers and bulbs in cotton; and exclusively ears in maize. Meanwhile, *H. zea* was found only in two sites, both in Viçosa, Southeastern Minas Gerais (Zona da Mata), and exclusively on maize ears (Figure 2). In both cases, *H. zea* was found co-occurring with *H. armigera* in the same fields (Figure 2).

Our results extend the knowledge on the presence and plant hosts of *H. armigera* in Brazil, with particular reference to Minas Gerais. This pest can now be considered widely dispersed in Southeastern Brazil, being present in economically important dicotyledon hosts, such as tomato, soybean, and cotton, as well as in monocotyledons, such as maize and sorghum [1,2,3,6]. Biological and socioeconomic factors can explain the fast spread of this pest insect in other regions of Brazil. Adults of *H. armigera* can migrate as far as 2000 km [7], and larvae are able to feed on at least 60 and 67 crop and non-crop plant species respectively [8]. In addition, the expansion of maize, soybean, and cotton crops in the previously infested regions could have facilitated the dispersal of this pest in Southeastern states.

One of the sampling sites (Paracatu in Northwestern Minas Gerais), where individuals of *H. armigera* were found, is located 80 km from the border of Goiás (Midwestern Brazil), where this pest was first reported in Brazil [1]. Besides both regions having extensive cultivation of soybean and cotton, massive shipments, especially of agricultural products, are taken from midwestern Brazil across Minas Gerais towards the coastal ports (to the states of Rio de Janeiro and Espírito Santo) for export. *Helicoverpa armigera* has recently been recorded in two sampling sites of chickpea crops in the province of Tucumán, Argentina [9]. Farmers from Chile, Uruguay and Paraguay have also reported lepidopteran attacks on several crops, and it is likely that those are also *H. armigera*, although no confirmed identification is yet available [10].

In all soybean and cotton sampling sites, *H. armigera* was the only *Helicoverpa* species found. In turn, *H. zea* was found on maize crops from Viçosa region, where mixed infestations with *H. armigera* were observed. Note that maize was not sampled in the other regions since there were no other crops at the time of sampling, so we cannot preclude the possibility that mixed infestations also occurred there. The fact of mixed infestations between these two species is very concerning due to the possibility of hybridization between these species in field conditions. PCR-RFLP is commonly used to identify these species yet cannot detect hybrids such as those that might arise between these two *Helicoverpa* species. For this reason, we cannot exclude the possibility that some of the insects we sampled are actually hybrids. The close relationship and high genetic similarity between *H. armigera* and *H. zea* is reflected in shared morphological and behavioural features [11]. For example, *Helicoverpa* species share the same chemical compounds in sex pheromones, hence allowing male *H. armigera* moths to be attracted by sex pheromones produced by female *H. zea* moths [12]. Under laboratory conditions, *H. armigera* and *H. zea* mate and produce fertile offspring [13]. However, hybridization under controlled conditions does not necessarily mean that the same is happening under field conditions. Nevertheless, this could lead to heterosis, that may present serious risks for crops, with the need of new strategies of control [14].

A further concern is that many of the *H. armigera* larvae found in this study were found in Bt-soybean and Bt-cotton crops. The rapid dispersion and the possibility of the development of resistance to insecticides and Bt crops of *H. armigera* mean that this pest may represent a major threat to Brazilian agriculture. In South-American countries, efforts have recently begun to monitor pest species and resistance to insecticides and Bt [10,15], however there is still a long path to obtain complete and adequate screenings to monitor and contain insecticide resistance.

## 4. Conclusions

Our field study indicates that *H. armigera* is present and established in different regions and host plants from Minas Gerais, and that it can co-occur with its close relative, *H. zea.* Since many populations of *H. armigera* around the world have acquired resistance to chemical pesticides and Bt crops [16], it is possible that the populations that originally invaded Brazil were already resistant. Due to these facts, the development of monitoring strategies for this pest and alternative control methodologies based on biological control may be key tools for future management of *H. armigera* in Brazilian crops.
